# SARS-CoV-2: Evolution and Emergence of New Viral Variants

**DOI:** 10.3390/v14040653

**Published:** 2022-03-22

**Authors:** Verónica Roxana Flores-Vega, Jessica Viridiana Monroy-Molina, Luis Enrique Jiménez-Hernández, Alfredo G. Torres, José Ignacio Santos-Preciado, Roberto Rosales-Reyes

**Affiliations:** 1 Unidad de Investigación en Medicina Experimental, Facultad de Medicina, Universidad Nacional Autónoma de México, Mexico City 06726, Mexico; v.roxana.flores.vega@gmail.com (V.R.F.-V.); jessvmm21@outlook.com (J.V.M.-M.); joseignaciosantos56@gmail.com (J.I.S.-P.); 2 Escuela de Ciencias de la Salud, Campus Coyoacán, Universidad del Valle de México, Calzada de Tlalpan 3000, Alcaldía Coyoacán, Mexico City 04910, Mexico; luis.jimenezh@uvmnet.edu; 3 Department of Microbiology and Immunology, University of Texas Medical Branch, Galveston, TX 77555, USA; altorres@utmb.edu

**Keywords:** SARS-CoV-2, COVID-19, viral variants, immune response

## Abstract

The severe acute respiratory syndrome coronavirus 2 (SARS-CoV-2) is the etiological agent responsible for the coronavirus disease 2019 (COVID-19). The high rate of mutation of this virus is associated with a quick emergence of new viral variants that have been rapidly spreading worldwide. Several mutations have been documented in the receptor-binding domain (RBD) of the viral spike protein that increases the interaction between SARS-CoV-2 and its cellular receptor, the angiotensin-converting enzyme 2 (ACE2). Mutations in the spike can increase the viral spread rate, disease severity, and the ability of the virus to evade either the immune protective responses, monoclonal antibody treatments, or the efficacy of current licensed vaccines. This review aimed to highlight the functional virus classification used by the World Health Organization (WHO), Phylogenetic Assignment of Named Global Outbreak (PANGO), Global Initiative on Sharing All Influenza Data (GISAID), and Nextstrain, an open-source project to harness the scientific and public health potential of pathogen genome data, the chronological emergence of viral variants of concern (VOCs) and variants of interest (VOIs), the major findings related to the rate of spread, and the mutations in the spike protein that are involved in the evasion of the host immune responses elicited by prior SARS-CoV-2 infections and by the protection induced by vaccination.

## 1. Introduction

Severe acute respiratory syndrome coronavirus 2 (SARS-CoV-2) is a highly transmissible RNA virus that causes “coronavirus disease 2019” (COVID-19). This emerging disease is transmitted by small droplets, or aerosols, expelled from infected individuals during person-to-person contact [1]. After infection, the first symptoms of viral infection are presented between 2 and 14 days, with major frequency occurring between 3 to 7 days [2]. Some COVID-19 symptoms are shared with those observed during influenza virus infections, e.g., headache, dry cough, sore throat, runny nose, nasal congestion, fever, myalgia, hypoxia, dyspnea, and, in some cases, diarrhea [3,4]. Particularly in COVID-19, the respiratory capacity of infected individuals decreases rapidly, leading to the development of pneumonia, cardiac injury, sepsis, and multi-organ dysfunction [1,3]. The rapid dissemination of SARS-CoV-2 across the countries around the world has been attributed to person-to-person contact and failure to promote the use of face masks or implementation of sanitary measures, but also due to limited access to vaccines against SARS-CoV-2. Additionally, the emergence of new viral variants of SARS-CoV-2 has reduced the efficiency by 28.2-fold of new and licensed vaccines to combat COVID-19. As a result of these variants, medical treatments that involve monoclonal antibodies have also been compromised.

SARS-CoV-2 is an enveloped, single-strand RNA virus, belonging to the coronavirus (CoV) family [3]. The viral genome is composed of approximately 30,000 nucleotides [5,6], with six functional open reading frames (ORFs) and four surface proteins: spike protein (S), the small envelope protein (E), the membrane protein (M), and the nucleocapsid protein (N) [3,7]. The S protein is a homo-trimeric glycoprotein that is localized on the viral envelope [8] and is cleaved by furine-like proteases, forming S1 and S2 subunits [9]. The S1 subunit contains an N-terminal domain (NTD) and a receptor-binding domain (RBD) that is responsible for the virus binding to the angiotensin-converting enzyme 2 (ACE2) receptor on the target host cell [4]. The S2 subunit carries out the fusion of the viral envelope with the host cell membrane [4,7,10]. The E protein is required for virion production and the M protein is involved in the virion assembly and budding, while the N protein is associated with the protection of the viral RNA inside the virion [7,10].

The high rate of viral replication, dissemination, and prevalence is associated with the emergence of new viral variants because these properties are associated with the acquisition of mutations in their genome. The mutagenesis events, particularly in the S1 subunit of the spike protein, can enhance its pathogenicity, infectivity, and dissemination [11,12]. In this work, we describe the classification used by the World Health Organization (WHO), Phylogenetic Assignment of Named Global Outbreak (PANGO), Global Initiative on Sharing All Influenza Data (GISAID), and Nextstrain, an open-source project to harness the scientific and public health potential of pathogen genome data to define the chronological emergence of new variants of SARS-CoV-2, which are classified as variants of concern (VOCs) and variants of interest (VOIs), as well as discussing the ability of each variant to evade the humoral immune response. We also describe the role of emergent viral variants in the evasion of protective immunity induced by prior exposition to SARS-CoV-2 or by the immunity induced by current vaccines.

## 2. Emergence and Classification of New Viral Variants

The high rate of viral replication is associated with the emergence of new viral variants that usually incorporate mutations in their spike protein [11,12]. Viral variants increase the efficiency of viral transmission, cell tropism, and pathogenicity, and escape immune recognition [13]. The high rate of mutation makes necessary a viral genome classification into lineages, groups, or clades. The WHO proposes a viral classification through the use of the Greek alphabet [14]. Other common nomenclature systems included those recommended by the GISAID [15], PANGO [16], and Nextstrain [17]. To identify variants that present a higher health risk, the WHO describe two major types of viral variants: variants of concern (VOC) and variants of interest (VOI) (Table 1). Thus, Alpha, Beta, Gamma, Delta, and Omicron are defined as VOCs, in contrast, the variants Lambda and Mu are classified as VOIs. Finally, the variants AZ.5 (formerly tracker under parent lineage B.1.1.318), C.1.2, B.1.617 (former VOIs: Kappa: B.1.617.1), B.1.630, and B.1.640 are variants under monitoring (VUMs) [14]. The Centers for Disease Control and Prevention (CDC) define VOCs as those with increased transmissibility, virulence, severity in the symptoms of disease (e.g., increased rate of hospitalization and deaths), decreased efficiency of antibodies (neutralization and diagnosis), and reduced effectiveness of treatments and protection induced by available vaccines. In contrast, viral variants with mutations that change the receptor binding affinity—increase the transmissibility (high community transmission) and the severity of the disease produced, affecting the affinity of the antibodies by the spike protein, and favoring the immune escape—and the effectiveness of the current diagnosis are those considered as VOIs. The PANGO nomenclature is designated to identify the current circulating lineages [16]. The classification, suggested by Rambaut et al., 2020 [18], uses a lineage name that begins with a letter A or B, and then the lineages that begin with the letter A are directly related with the Wuhan/WH04/2020 variant, and the lineages that begin with the letter B are associated with the Wuhan-Hu-1 variant. The new SARS-CoV-2 lineages descending from a lineage A or B are assigned with a numerical value (e.g., lineage A.1 or B.2). The specific lineage designation needs to follow this set of conditions: (1) Every descendant lineage needs to show phylogenetic evidence of emergence from an ancestral lineage of a different geographical population. To assign phylogenetic evidence, the lineage needs to follow these criteria: (a) share one or more nucleotide differences from the ancestral lineage, (b) it should comprise at least five genomes with >95% of the genome sequenced, (c) genomes belonging to the new lineage must exhibit at least one shared nucleotide change among them, and (d) a bootstrap value >70% for the new lineage node is required. (2) The identified lineages in step 1 could be used as ancestors for new virus lineages that emerge in another geographical area (e.g., A.1.1). (3) Thus, this procedure might proceed for a maximum of three sublevels (e.g., A.1.1.1). If a new descendant lineage emerges, a letter will be assigned (e.g., A.1.1.1.1 would become C.1) [18]. With this nomenclature, the variants of concern were designated as B.1.1.7, B.1.351, P.1, B.1.617.2, and B.1.1.529 (Table 1) [16]. GISAID classifies the new SARS-CoV-2 variants into clades. A clade is defined by the statistical distribution of the viral genomes distance into phylogenetic clusters [19], followed by the merging of smaller lineages into major clades. Thus, viral variants are classified into eight high-level phylogenetic groups from an early split of S and L, and then by an evolution of L into V and G, and later of G into GH, GR, and GV, and, more recently GR into GRY [15,20]. Finally, Nextstrain classifies SARS-CoV-2 into 14 major clades: 19A, 19B, and 20A-20L. A clade is created when a new variant reaches a global frequency of 20% at any given time. The clade name is associated with the year in which a new variant emerges, and in this case, the clade name for the new viral variant uses the next letter in the alphabet. To define a new clade, the variant should have two new mutations related to its parent major clade. Thus, the major clades by year are defined by their emergence and a letter, e.g., 19A, 19B, or 20A [21]. Current clades that are circulating worldwide are 19A (from Asia: China/Thailand), 19B (from Asia: China), 20A (from North America/Europe/Asia: USA, Belgium, and India), 20B (from Europe: UK, Belgium and Sweden), and 20C (from North America: USA) (Table 1) [21,22].

## 3. Variants of Concern and Variants of Interest

The WHO defined VOCs, VOIs, and VUMs in function of their genome mutations, their properties of spreading between susceptible hosts and the disease severity produced, and by the evasion of the immune response elicited by current available vaccines and by the therapeutic treatments with convalescent plasma or by the use of therapeutic monoclonal antibodies. 

### 3.1. Alpha Variant

**Figure 1:**
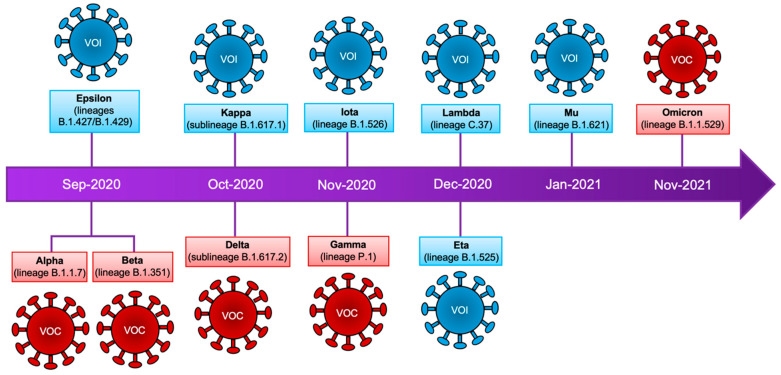
SARS-CoV-2 variants. Timeline that summarizes the emergence of SARS-CoV-2 variants. Classification according to the WHO is shown as well as the lineages of the mutagenic profile. VOC: variants of concern, VOI: variants of interest.

This variant (lineage B.1.1.7) was identified in September 2020 in the United Kingdom (Figure 1, Table 1) [14]. This variant is characterized by the presence of nine mutations in the spike protein (Table 2) as compared with the original virus isolated in China [23]. The introduction of mutations in the Q493N and Q498Y positions of the spike protein increases the viral binding to the cell host ACE2 receptor [24,25]. In contrast, the H69del and V70del mutations in the S1 subunit at the N-terminal domain of the spike protein are associated with the evasion of the host immune response (reduced efficacy of neutralizing antibodies and convalescent plasma) [26]. The P681H mutation is required to mediate resistance to the antiviral effect of interferon-β (IFNβ) on lung epithelial cells, in contrast with the spike protein from Wuhan strain; additionally, this mutation confers the ability to evade the host immune response [27]. In December 2020, an additional mutation (E484K) was detected during the Bamlanivimab monotherapy treatment on high-risk patients infected with the Alpha variant [28]. It has been observed that these nine mutations increased the viral transmissibility [29,30,31], as well as the risk of hospitalization and the rate of case fatality [32]. Importantly, the mutation in D614G in the spike protein has become the dominant mutation in all variants of SARS-CoV-2 detected worldwide to date (Table 2) [33,34]. Interestingly, the Pfizer-BioNTech (BNT162b2) vaccine has been shown to be 89.5% effective against the Alpha variant after receiving two doses [35].

### 3.2. Beta Variant

This variant (lineage B.1.351) was identified in September 2020, in South Africa (Figure 1, Table 1) [36]. This variant contains nine mutations in the spike protein (Table 2) as compared with the original SARS-CoV-2 virus [23]. Three mutations (K417N, E484K, and N501Y) in the RBD domain promote the viral escape of immune recognition and enhance 19 times their affinity for the cellular receptor ACE2 [37]. In addition, this viral variant spreads faster between young and healthy individuals, who are more likely to develop a severe disease [36]. This variant also presents an increased transmissibility rate, risk of hospitalization (31%), and death (17.7%) [38]. The incorporation of new mutations in this variant compromises the protection efficacy provided by the available vaccines. Indeed, the Pfizer-BioNTech vaccine was found to be 75% effective against this viral variant after two doses and 97.4% effective against severe or fatal disease [35]. The Novavax vaccine showed 86% efficacy after two doses against this variant, and, in contrast, this vaccine showed a reduced efficacy (60%) against the Beta variant [39]. Importantly, the ChAdOx1 nCoV-19 vaccine from Oxford-AstraZeneca showed low efficacy (10%) after two doses against this variant [40].

### 3.3. Gamma Variant

This variant (lineage P.1) was identified in November 2020, in Japan and Brazil (Figure 1, Table 1) [14]. This variant arose from lineage B.1.1.28 and its spike protein has 12 mutations (Table 2) [23]. Particularly, L18F, K417N/T, E484K, N501Y, and D614G were also identified in the RBD domain of the spike protein from the Beta variant (Table 2) [41,42]. These mutations have been shown to have important implications, both in the transmissibility of the virus and the rate of reinfection. In addition, these mutations reduce the efficacy of monoclonal antibodies therapy [43]. The plasma of convalescent patients and the sera of immunized individuals showed a reduction in their neutralizing activity against this variant [44,45]. The efficacy of two doses of Pfizer or Oxford-AstraZeneca vaccines against this variant is low (~50% of efficacy) [46]. In the case of CoronaVac, this vaccine has an efficacy of 37–59% [47,48]. In contrast, Moderna (mRNA-1273/Moderna) showed a reduced efficacy of protection (61%) after two doses [49].

### 3.4. Delta Variant

**Figure 2:**
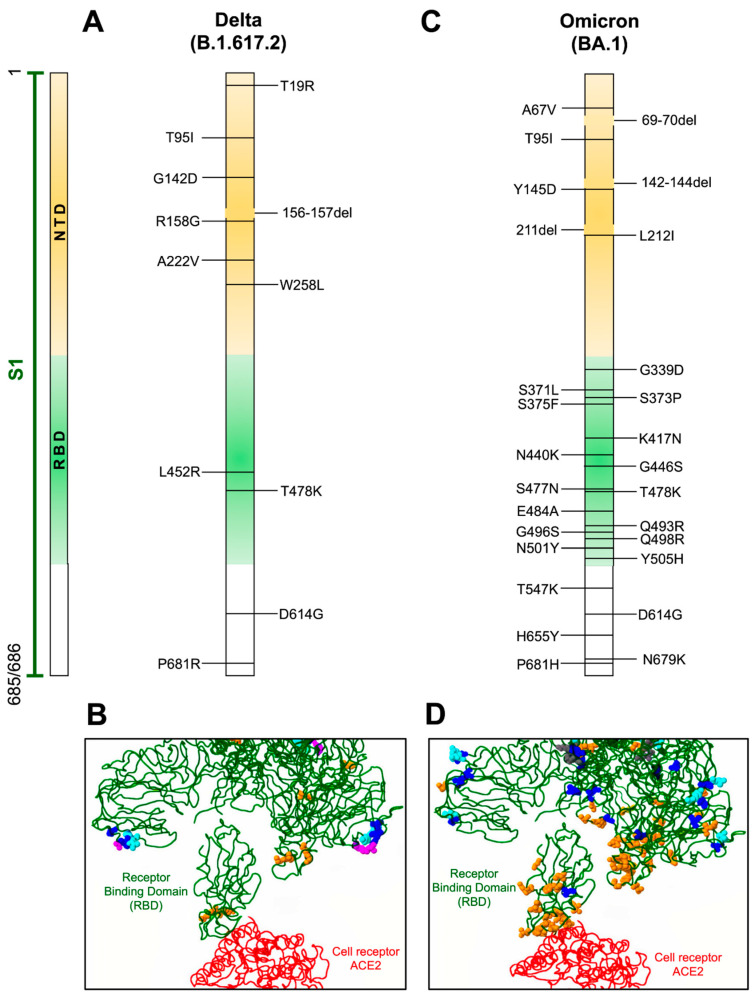
Mutations in Delta and Omicron variants. (**A**,**C**) Localization of defined mutations in the S1 domain and RBD of the spike protein of Delta and Omicron SARS-CoV-2 variants. (**B**,**D**) Model of mutations associated with the RBD domain (green) of the spike protein in interaction with the ACE2 receptor (red). Amino acid changes occurring more than one hundred times are indicated in blue, amino acids involved in direct interaction with ACE2 or associated with antigenicity are displayed in orange, potential amino acids involved in glycosylation are depicted in magenta, and amino acids inserted or deleted are indicated in cyan. GenBank access for the Delta variant is QWK65230.1 and for Omicron is OM095411.1. Modeling of the RBD domain with ACE2 was evaluated with the CoVsurver mutations app available on GISAID web page [57].

This variant (sub-lineage B.1.617.2) was identified in October 2020, in India (Figure 1, Table 1) [14]. This variant has eleven mutations in the spike protein (Table 2) and belongs to the sub-lineage B.1.617. The Delta variant is characterized as highly transmissible and has spread worldwide between fully vaccinated as well as in unvaccinated individuals [50]. This variant lacks the mutations at positions 501 and 484 in the RBD domain of the spike protein that is associated with evasion of the neutralizing activity of antibodies (Table 1, Figure 2A). The Delta variant became globally dominant by June 2021. Mutations in the RBD domain (Figure 2A,B) abrogate the binding of some monoclonal antibodies. The antibody Bamlanivimab had reduced binding to RBD but did not improve ACE2 binding [51,52]. In addition, sera collected from convalescent individuals were fourfold less potent against the Delta variant relative to the Alpha variant [52]. During in vitro experiments, the sera from immunized individuals with the Pfizer-BioNTech vaccine showed a binding reduction of 2.4-fold in a pseudovirus binding assay. In addition, neutralizing antibodies titers for the Pfizer-BioNTech elicited plasma were reduced 4.1-fold with the Delta+ variant (with K417N additional mutation in B.1.617.2 sub-lineage), and the plasma from individuals immunized with the Moderna vaccine showed a reduction of 2.6- and 9.5-fold to Delta and Delta+ variants, respectively. In contrast, sera from individuals immunized with the Johnson and Johnson (Ad26.COV2) vaccine showed a reduction of 2.4- and 3.5-fold with Delta and Delta+, respectively [51]. The reduced efficacy of these vaccines are due in part to the mutations L452R and T478K, which are localized in the antigenic site I of the RBD (Figure 2B) [53,54,55]. Thus, neutralizing antibodies have reduced binding to the spike protein. Likewise, the P681R mutation enhances the cleavage of the full-length spike to S1 and S2 subunits, leading to increased infection via binding to the ACE2 receptor on the target cells [56]. 

### 3.5. Epsilon Variant

This variant was identified in September 2020 in California, USA (Figure 1, Table 1) [23] and encompasses the B.1.427 and B.1.429 lineages [5,58]. Mutations in W152C and S131 of the spike protein increase the infectivity of the variant [58]. In addition, the mutation in L452R increases the interaction with ACE2 to enhance dissemination (24%) [59]. In particular, it has been demonstrated that the efficacy of neutralization of the murine leukemia virus pseudotyped system expressing SARS-CoV-2 proteins by Moderna vaccine-elicited plasma was reduced 2.4-fold, whereas a 2.3-fold reduction was observed with the Pfizer-BioNTech vaccine [60]. In addition, the potency of neutralization by plasma from individuals vaccinated with the Pfizer-BioNTech vaccine was reduced 2.9-fold and with convalescent plasma 3.4-fold [60].

### 3.6. Eta Variant

This variant (lineage B.1.525) was detected worldwide in December 2020 (Figure 1, Table 1) [14] and has six mutations and three deletions in the spike protein (Table 2) [23]. The E484K mutation increases its infectivity and contributes to the reduction of the effectiveness of neutralizing antibodies induced by SARS-CoV-2 natural infection, monoclonal antibodies, and by vaccination with Pfizer-BioNTech [61,62]. In addition, the E484K mutation is associated with an increase of infecting cells expressing ACE2 [63]. Finally, the Q677H mutation is also responsible for its increased transmissibility and syncytia formation [64]. The Pfizer-BioNTech vaccine works well against this variant [65]. Currently, reports show that African countries such as Nigeria and Sudan have a high prevalence of this variant [63].

### 3.7. Iota Variant

This variant (lineage B.1.526) was identified in November 2020 in New York State, USA (Figure 1, Table 1) [23]. Two versions of the B.1.526 variant were identified, with both variants having the D614G and A710V mutations. Importantly, the mutations L5F, T95I, and D253G have not been previously reported in other variants (Table 2). In particular, the B.1.526 (S477N) variant was efficiently neutralized with the monoclonal antibody Regeneron. In addition, convalescent-phase sera from individuals vaccinated with Pfizer-BioNTech vaccine retain their ability to neutralize the B.1.526 (S477N) variant. In contrast, the B.1.526 variant (E484K) showed a 3.5-fold reduction in titer of the convalescent plasma and sera from vaccinated individuals [66]. Additionally, sera of individuals vaccinated with the Moderna vaccine showed an efficacy of 88% in the neutralization of the B.1.526 lineage [67].

### 3.8. Kappa Variant

This variant (sub-lineage B.1.617.1) was detected in October 2020 in India (Figure 1, Table 1) [68]. This variant was rapidly replaced by the Delta variant [69], therefore, B.1.617.1 sub-lineage is considered as the predecessor of the Delta variant [70]. This variant possesses six mutations in the RBD domain that conferred a higher infectivity rate (Table 2) [71,72]. The mutation in E484Q is critical for the increased binding of the spike protein to ACE2 [69,71]. The P681R mutation is considered one of the most significant ones because it is located adjacent to the furin cleavage site, which facilitates the virus entry into the target cell [70]. In contrast, the L452R mutation stabilizes the interaction between spike and ACE2 to increase their transmissibility; moreover, this mutation is also associated with resistance to neutralizing antibodies [73,74]. Significantly, individuals immunized with the Pfizer-BioNTech and Moderna vaccines have reduced neutralization titers against the Kappa variant (6.8 times lower), and even so, all vaccinated individuals have the ability to neutralize infection by this variant [75]. In addition, it has been reported that immune escape in convalescent individuals and those who are vaccinated (BNT162b2 and mRNA-1273) is specifically related to the L452R and E484Q mutations. Although the vaccine protects against the Kappa variant, it only does it for a reduced period (protects for three months after the second dose) [76,77]. Likewise, several studies have shown a reduction in neutralizing antibodies, of 3.9-fold, from sera of convalescent individuals, 2.7-fold in sera from people vaccinated with the Pfizer-BioNTech vaccine, and 2.6-fold in individuals vaccinated with the Oxford-AstraZeneca vaccine. Nevertheless, while a reduction in neutralizing antibodies exists, no overall evasion of the immune response has been reported [78].

### 3.9. Lambda Variant

The Lambda variant (C.37 lineage) was first identified in December 2020 in Perú (Figure 1, Table 1) [23]. This variant has six mutations in the spike protein and a deletion of seven amino acids in the N-terminal domain (RSYLTPGD246–252del) (Table 2) [23,79,80]. In particular, 246–252del, L452Q, and F490S mutations mediate resistance to the neutralization activity of the sera obtained from vaccinated individuals. Mutations in T76I and L452Q are associated with increased viral infectivity [81]. In addition, the T859N mutation has been associated with a decrease in the neutralizing activity of monoclonal antibodies [82]. The L452Q and D614G mutations increased the viral replication and dissemination [79,82]. The serum from individuals vaccinated with the Moderna vaccine was 2.3-fold less effective in their neutralization activity, while serum from individuals vaccinated with the Pfizer-BioNTech reduced their efficacy threefold [80]. 

### 3.10. Mu Variant

The Mu variant (B.1.621 lineage) was first identified in Colombia on 11 January 2021 (Figure 1, Table 1) [14]. This variant has nine mutations in the spike protein (Table 2), five of these are shared with other VOCs (Table 2). The antibodies from convalescent individuals showed strong recognition to Alpha and Delta variants but decreased recognition to Beta and Mu variants [83]. In this context, two mutations (YY144–145TSN and E484K) are responsible for resistance to convalescent sera and also sera from vaccinated individuals [83]. The WHO reports that the Mu variant only represents 0.1% of cases worldwide, with a high rate of infection in Colombia (39%) and Ecuador (13%), while sporadic cases have occurred in some European countries [14]. The activity of sera from individuals vaccinated with the Pfizer vaccine was reduced 9.1-fold, while sera from convalescent patients was reduced 10.6-fold [84]. Moderna’s vaccine maintains a 45.8% protection with one dose and 90.4% with a second dose [85]. 

### 3.11. Omicron Variant

This variant (B.1.1.529 lineage) was identified in 11 November 2021 in Botswana, Africa (Figure 1, Table 1) [86]. This variant has more than 30 mutations in the spike protein (B.1.1.529/BA.1) (Table 2, Figure 2C,D). So far, Omicron has yielded at least three genetically related viral sub-lineages (BA.1, BA.2, and BA.3) that diverged from the B.1.1.529 lineage [86]. Many of these changes have been found in the Delta and Alpha variants (Table 2) [87], which are linked to the increase in the infectivity and the ability to evade infection-blocking antibodies and antibodies induced after vaccination [35,51,53,54,55,67,88]. The significant number of mutations present in the spike protein has been associated with a high rate of transmissibility, immune resistance, increased risk of reinfection [89], decreased lung infectivity, and lower pathogenicity compared with Delta variant [90]. In addition, Omicron does not induce cell syncytia on A549-ACE2 cells [91]. The emergence of this new variant is associated with viral evolution in immunosuppressed individuals [87]. As mentioned, the Omicron variant has mutations described in other VOCs, such as N501Y, which is present in the B.1.1.7, B.1.351, P.1, and P.3 lineages, and is associated with improved binding of the RBD domain of the spike protein to the ACE2 receptor, resulting in higher transmissibility [92,93]. It also shares the K417N and E484K mutations (E484A in the Omicron variant) with the B.1.351 and P.1 lineages, which are linked to evasion of the immune response [62]. Nevertheless, some studies have shown that the Omicron variant has a lower replication rate in lung cells than the B.1.617.2 lineage [94]. In addition, Omicron is characterized by its ability to evade the humoral immune response in individuals who have a full vaccination scheme (including the booster dose) [67,88], and therefore, Omicron is considered more infectious (2.7–3.7 times higher) than the Delta variant [95]. Mutations in spike protein alter the local conformation, charge, and hydrophobic microenvironments that also decrease the host humoral immune response and the neutralization efficacy provided by the monoclonal antibody mAb S309 (related to mAb sotrovimab) and mAb CR3022 [96,97,98]. The efficacy of the Pfizer vaccine against this variant decreases 28.2-fold, however, a third dose increases the effectiveness of the Pfizer vaccine 23.4-fold [67,88], suggesting that the third boost of the Pfizer vaccine can efficiently neutralize this variant. In fact, a third dose of the Pfizer or Moderna vaccine schemes increases the efficiency of neutralizing antibodies, as well as the combination of the Johnson and Johnson (Ad26.COV2) vaccine with a booster of Pfizer, which also crucially increases the activity of neutralizing antibodies against Omicron [88].

## 4. Concluding Remarks

The emergence of SARS-CoV-2 in late 2019 has made the health systems of all the countries around the world become immersed in important public health issues to combat the virus infection and its spread. The emergence of new viral variants that incorporate novel mutations in their genome has caused an increase in their infectivity, spread, and prevalence. The incorporated mutations in the genome of the new viral variants caused organizations and online platforms like WHO, PANGO, GISAID, and Nextstrain to classify these mutations in variants, clades, or lineages, and identify their global spread and evaluate the potential of emergence of other new viral variants. Most of the new viral variants incorporate new mutations in the S1 domain of the spike protein, increasing its interaction with ACE2 and eventually decreasing the efficacy of neutralizing antibodies (monoclonal antibodies, convalescent plasma, and sera from vaccinated individuals). In particular, Omicron is an emergent variant that shows more than thirty mutations in the spike protein. These changes increase its interaction with ACE2 and these amino acid sequence changes also impact the loss of epitopes recognized by the neutralizing antibodies. Nevertheless, current licensed vaccines work well—antibodies induced with two doses of the Pfizer-BioNTech vaccine efficiently controlled the infection by Alpha, Beta, Eta, Iota, and Kappa variants, and even neutralized the Omicron variant with a third booster dose. Some variants, such as Gamma, Delta, Epsilon, Lambda, and Eta, have shown resistance to the neutralization activity of antibodies induced by vaccination. It is evident that a complete vaccination scheme could increase the production of neutralizing antibodies and, also, it would be desirable to produce vaccines that neutralize the mutations associated with evasion of the humoral immune response. Finally, the currently licensed vaccines seem to work well because they reduce the risk of severe infection and the mortality rate. Epidemiological surveillance is a key strategy to determine the emergence of new viral variants and to identify their spread and rate of fatality. The development of a better version of the current vaccines or a pan-coronavirus vaccine, in combination with the identification of better monoclonal antibody treatments, could help decrease the emergence of new viral variants and, thus, reduce the mortality and morbidity caused by the SARS-CoV-2 infection.

## 5. Limitations of This Study

(a) This study was conducted by revision of the published scientific literature, and we reviewed several papers that studied variants defined by WHO or PANGO, but we have not included sub-lineages that are not defined as VOCs and VOIs, and as such, their contribution to the global infection may not be included in this study; (b) the fast-growing body of knowledge and the rapid emergence of new viral variants limits the scope of this study and such available information is not included here. Nevertheless, the strengths of our work are (a) definition of viral variants by available platforms (WHO, PANGO, GISAID, and Nextstrain), (b) the chronological emergence of viral variants, and (c) description of mutations associated with the viral spread and evasion of the host immune response.

## Figures and Tables

**Table 1:** Classification of SARS-CoV-2 viral variants identified worldwide.

WHO	PANGO (Lineage)	GISAID (Clade)	Nextstrain (Clade)	Country of Identification
Alpha	B.1.1.7	GRY	20I (V1)	UK
Beta	B.1.351	GH/501Y.V2	20H (V2)	South Africa
Gamma	P.1	GR/501Y.V3	20J (V3)	Japan/Brazil
Delta	B.1.617.2	G/478K.V1	21A	India
Epsilon	B.1.427/B.1.429	GH/452R.V1	21C	USA
Eta	B.1.525	G/484K.V3	21D	Multiple countries
Iota	B.1.526	GH/253G.V1	21F	USA
Kappa	B.1.617.1	G/452R.V3	21B	India
Lambda	C.37	GR/452Q.V1	21G	Perú
Mu	B.1.621	GH	21H	Colombia
Omicron	B.1.1.529	BR/484A	21K	Africa

WHO, World Health Organization; PANGO, Phylogenetic Assignment of Named Global Outbreak Lineages; GISAID, Global Initiative on Sharing All Influenza Data; UK, United Kingdom; USA, United States of America.

**Table 2:** Variants of SARS-CoV-2.

Variant (Lineage or Sub-Lineage)	Mutations in Spike	
	S1	S2
Alpha (B.1.1.7)	69–70del, 144del, N501Y, A570D, D614G, P681H	T716I, S982A, D1118H
Beta (B.1.351)	L18F, D80A, D215G, 241–243del, K417N, E484K, N501Y, D614G	A701V
Gamma (P.1)	L18F, T20N, P26S, D138Y, R190S, K417N/T, E484K, N501Y, D614G, H655Y	T1027I, V1176F
Delta (B.1.617.2)	T19R, T95I, G142D, 156del, 157del, R158G, L452R, T478K, D614G, P681R	D950N
Epsilon (B.1.427, B.1.429)	S13I, W152C, L452R, D614G	
Eta (B.1.525)	Q52R, A67V, H69del, V70del, Y144del, E484K, D614G, Q677H	F888L
Iota (B.1.526)	L5F, T95I, D253G, S477N, E484K, D614G	A701V
Kappa (B.1.617.1)	T95I, G142D, E154K, L452R, E484Q, D614G, P681R	Q1071H
Lambda (C37)	G75V, T76I, 246–252del, L452Q, F490S, D614G	T859N
Mu (B.1.621)	T95I, Y144S, Y145N, R346K, E484K, N501Y, D614G, P681H	D950N
Omicron (B.1.1.529/BA.1)	A67V, 69del, 70del, T95I, 142del, 143del, 144del, Y145D, 211del, L212I, G339D, S371L, S373P, S375F, K417N, N440K, G446S, S477N, T478K, E484A, Q493R, G496S, Q498R, N501Y, Y505H, T547K, D614G, H655Y, N679K, P681H	N764K, D796Y, N856K, Q954H, N969K, L981F

Mutations in the spike protein of SARS-CoV-2 were obtained from the Swiss Institute of Bioinformatics [23].
